# Size, Rarity and Charisma: Valuing African Wildlife Trophies

**DOI:** 10.1371/journal.pone.0012866

**Published:** 2010-09-22

**Authors:** Paul J. Johnson, Ruth Kansky, Andrew J. Loveridge, David W. Macdonald

**Affiliations:** Wildlife Conservation Research Unit, Zoology Department, Recanati-Kaplan Centre, University of Oxford, Oxford, United Kingdom; University of Kent, United Kingdom

## Abstract

We explore variation in the prices paid by recreational hunters of trophy animals in Africa and its possible causes, including perceived rarity. Previous work has raised the possibility that extinction can result if demand rises fast enough as a species becomes rarer. We attempt to disentangle this from other inter-correlated influences affecting price. Species with larger body sizes and larger trophies were more valuable. Value increased less steeply as a function of size for bovids than for felids and the effect was consistent across countries. Power laws, ubiquitous in physical and social systems, described the trends. The exponent was approximately 0.4 for bovids, compared with approximately 1.0 for felids. Rarity (as indexed by IUCN score) influenced the value of bovid trophies – price was higher for species in categories denoting higher global threat. There was substantial variation in price among and within families not explained by either size or rarity. This may be attributable to a ‘charisma’ effect, which seems likely to be a general attribute of human perceptions of wildlife. Species where prices were higher than predicted by size or rarity are ranked high in published accounts of desirability by hunters. We conclude that the valuation of these species is explicable to a large extent by body size and perceived rarity, and that differences in valuation between taxonomic groups are related to less easily quantified ‘charisma’ effects. These findings are relevant for conservationists considering the threat status of species exploited in open access markets, and where license quotas are adjusted in response to changes in perceived rarity.

## Introduction

In trophy hunting a conventional market exists, whereby participants pay a fee to hunt an animal and to keep a trophy from it. From an economic perspective price is an unambiguous index of desirability [1]. Wildlife conservationists differ in their assessment of trophy hunting, particularly its economic impact and potential for contributing to conservation [2], [3]. An important aspect of both is the value of trophies. We explore variation in trophy prices and its possible causes both to illuminate generalities about human valuation of wildlife and to assess the importance of the patterns for the conservation of the hunted species.

Several inter-related variables are candidates for predicting trophy prices. More abundant taxa are expected to be cheaper than rare taxa. Courchamp et al. [4] showed that the trophy price of hunted caprinids was related to an index of rarity based on International Union for Conservation of Nature (IUCN) listing. They speculated that an anthropogenic Allee effect (AEE), where rarity fuelled demand disproportionately, could have serious conservation implications. If demand rises fast enough with rarity, it may remain economically viable to hunt a species to extinction. In the absence of this Allee effect, standard economic theory predicts that exploitation does not necessarily lead to extinction, as the cost of finding the last individuals should at some point exceed their market value (though this does not take account of inbreeding depression, or the effect of the social system of the species[5]).

Valuing rarity may be a general phenomena; Angulo et al. [6] showed that visitors to zoos were prepared to invest more time, or to endure more difficult conditions, to view enclosures labeled as, but not in fact containing, rare species. In the market for luxury goods, there is evidence that *perceived* rarity dominates quality in determining demand [7]. The effect of rarity on the price of hunting trophies is likely to depend crucially on two factors. First, whether demand for the trophy as a commodity is ‘elastic’, where demand changes more than proportionately with price, or ‘inelastic’, where demand is relatively unaffected by price. Milner-Gulland et al. [8] hypothesised that for the ‘big five’ (lion, leopard, elephant, rhino and buffalo) demand is inelastic compared with that for less charismatic species. The second important consideration is whether the hunting is ‘open access’ or not. If the resource is not open access, as it is not in this study, then a rational owner's optimal option for rarity-fuelled increased demand for the big five should be to increase the price, not to allow unsustainable hunting. However, in the presence of imperfect knowledge on sustainability and large incentives to overexploit, perceived rarity may still be hazardous.

The value of individual animals is likely to increase with size as large species are scarcer than small species. The relationship between size and rarity follows a power law [1]. Taking this into account, Peters (1983) drew attention to the increase in cost of hunting licences with game size (Peters 1983, p187), and the tendency for large species to be favoured both by governments funding conservation and by those concerned with animal welfare. For felids hunted for trophies in Africa, mean price increases proportionately with body size [9]. Size-price effects also incorporate handling costs: large carcasses are more expensive to process and transport.

There are a variety of other potential determinants of price, some of which vary geographically; buffalo trophies from Tanzania and Zimbabwe are considered to be superior, for example [10], and there are also differences among countries in pricing policy – in Tanzania market principles are not used to set quota sizes [2]. Our explorations account for these differences by including country identity in statistical models. Other effects are largely absorbed by the ‘rarity’ effect, the administration cost of hunting CITES listed species is one such.

Hunters' attitudes to hunted species are also likely to be affected by less easily quantified influences – for example, whether an animal is a predator [11]; fear and awe of predators is probably a deep-seated human disposition [12]. Batt [13] observed (in a sample of UK students) more positive attitudes to species with perceived similarity to humans. Psychological explanations of attitudes to animals, including ‘terror management’ and ‘mortality salience’ [13] may have particular significance for the attitude of hunters to hunted animals. In some cases, the difficulty and danger of hunting a species, and therefore the prestige of owing part of it as a trophy, are likely to contribute to its value to a hunter.

Morphology is generally similar within hunted families, and rarity and size are likely to be the major determinants of price. For bovids, we also ask if the size of a trophy, which indicates prestige among some of the hunting fraternity [14], is a better signal of value than is body mass. We estimate the form of the link between price and its predictors as evidence of how value is perceived, and we compare these relationships among hunted families. These functions have implications for both the psychology of valuation and for conservation. For example, the Allee effect proposed by Courchamp et al. [4] depends on the strength of the relationship between perceived rarity and price. We find that power laws with exponents of between c 0.3–1.00 link value and size, and that within bovids rarity adds to the trophy size effect. Value variation between families is likely to be linked to widespread attitudinal differences to animals.

## Results

### Family level effects

**Figure 1:**
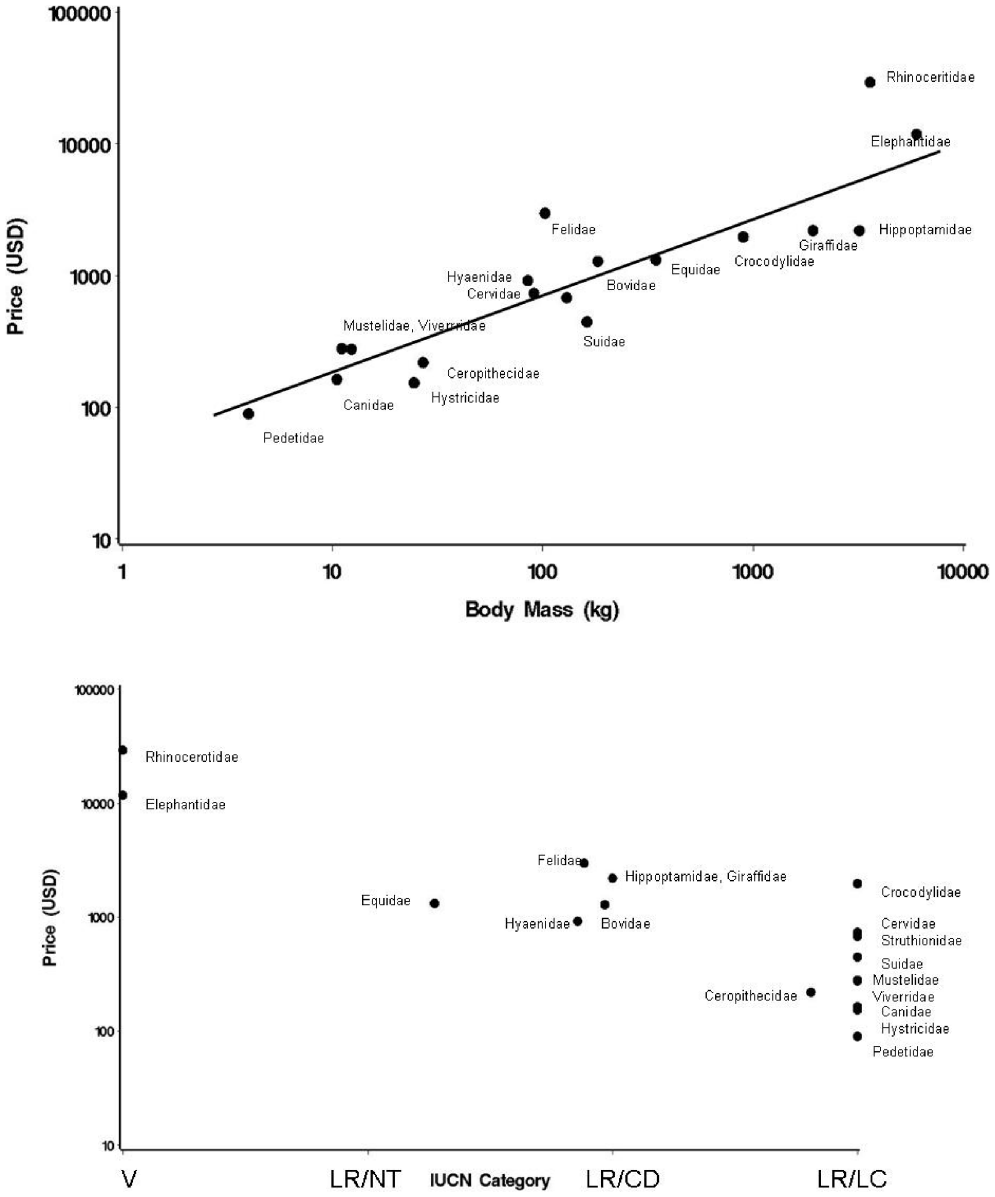
Family level effects on trophy prices. a) Mean price (MP) as a function of mean body mass (MBM). MP = 42.5*MBM ^0.62^, exponent CI 0.462 to 0.778, R^2^ = 80.5% b) MP and IUCN category. MP = e ^(11.1–1.31.IUCN)^, exponent CI −1.51 to −1.11) R^2^ = 73.7%. (V = vulnerable, LR/NT = Least risk, near threatened, LR/CD = Least risk, conservation dependent, LR/LC = Least risk, least concern). Family locations plotted as mean among ordinal categories.

Overwhelmingly (*w*>0.95), the best model for predicting mean values at the family level included country, (mean) IUCN score and (mean) body mass, with a power law for the body mass effect (R –squared = 80.2%). Country explained 10.9% of the variability in the response. Mean prices were significantly higher in Mozambique, Cameroon and South Africa, compared with the Central African Republic and Ethiopia (Tukey means separation procedure). Prices increased with body mass and declined with IUCN class (Figure 1).

The slope of the body mass effect was 0.39 (CI: +0.31to +0.47) implying a power law where value increased much less steeply than in proportion with the characteristic body mass of the family. The parameter estimate for IUCN class was −0.63 (CI: −0.81 to −0.46). Both predictors were influential in models adjusting for each other. Partial R-squared values for body mass and IUCN class in models adjusting only for country were 59.4% and 53.3%, respectively. In models where each was entered last of three predictors the values were 15.2% and 8.9%, respectively. Mass is therefore a marginally better predictor. If family price was summarized to a single datum across taxa within the family (figure 1) conclusions about the magnitude and direction of effects did not differ. The robustness of the effects was confirmed in separate analyses for each country. Slopes varied between 0.38 and 0.53 for body mass and between −1.0 and −0.20 for IUCN class.

A qualitative exploration of the SCI ‘remarks’ on hunted species in the different families partially supports the idea that deviation from the price/size relationship are related to hunters' perceptions of the family's ‘charisma’. Hippos, for example, with a large negative residual in figure 1a are said to be ‘not very difficult to hunt’ [14], and taken mostly for lion bait or to ‘fill out a collection’. Similarly, of the Suidae, also ranked low on this index, the SCI comments ‘Not a top game animal’ (warthog). Rhinos, with the second highest positive residual, are described as elusive and ‘challenging’. Felids, with the largest absolute value of residuals adjusting for both body size and IUCN class attract such remarks as ‘highly esteemed’ (lion) and ‘fine trophy’ (leopard). The SCI do not comment on some familes (eg Hyaenidae, Viverridae), though trophy records are kept.

### Species effects within families

Bovids hunted as game range from antelopes of less than 3 kg to Eland of up to 900 kg [15]. The points in figure 1 are crude summaries for some taxa. In spite of this, the mean price to hunt bovid species is well predicted by their size. We explore patterns within the groups represented by these points. For the five species of canid (three jackals and two foxes) in our data set, we found no link between body mass and price, or between price and any index of rarity. The same was true of the seven primates (five baboons and two monkeys). Jackals and some primates (Cercopithecids) are widely regarded as vermin. However, in the bovids and felids we found some consistent patterns in how species were valued depending on their size, rarity and trophy status.

### Bovidae

**Figure 2:**
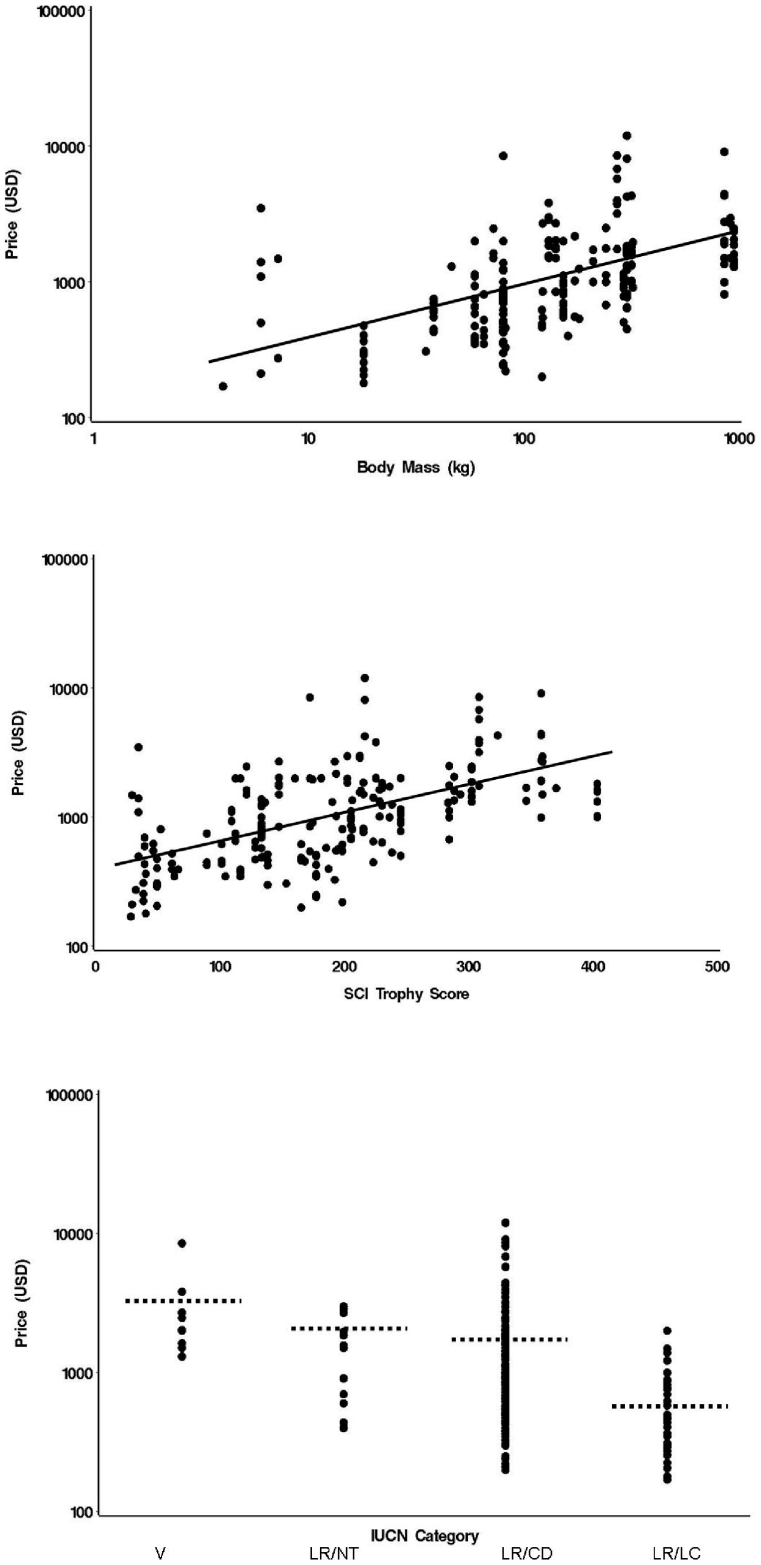
The influence of a) body mass BM, b) SCI index and c) IUCN category on mean price (MP) for bovid taxa within countries. (Dashed line denotes category means among taxa: V mean = $3112.2 (SE = 685.7), LR/NT $1640.9 (256.1), LR/CD $1481.7 (129.6), LR/LC $610.3 (78.4)).

All models included country (entered first) which was a useful predictor of price (partial R-squared 10.2%). Only two models were weighted non-trivially (with w>0.10, table 1). Both included body size, trophy size and IUCN score as well as country. A power law predicting price using both size and IUCN score was the best fit (r-squared = 51.9%, table 2). Trophy value increased less than in proportion with body mass and declined in proportion with IUCN score (figure 2). Trophy size was a better predictor than body mass, and IUCN score was about half as useful as body mass (table 2). All four predictors were nevertheless statistically significant irrespective of their order of entry to (sequential) statistical models. We fitted within country models to allow for the different taxa hunted within each – these gave the same direction and magnitude of effects.

**Table 1:** Evaluation of models for predicting bovid trophy price. All 26 models included country ID as blocking factor.

Parameters	AICc	Model	AICc diff	Evidence Ratio	Weight	Cumulative Weight
15	418.95	lnWT+SCI+lnIUCN	0.000	1.00	0.515	0.515
15	419.97	WT+SCI+lnIUCN	1.023	1.67	0.309	0.824
14	422.35	SCI+lnIUCN	3.400	5.47	0.094	0.918
15	424.98	lnWT+SCI+IUCN	6.027	20.36	0.025	0.944
15	425.30	WT+SCI+IUCN	6.351	23.94	0.021	0.966
14	426.12	lnWT+lnIUCN	7.174	36.12	0.014	0.980
14	427.06	SCI+IUCN	8.114	57.81	0.009	0.990
15	428.15	lnWT+lnSCI+lnIUCN	9.204	99.69	0.005	0.999
15	428.34	WT+lnSCI+lnIUCN	9.393	109.55	0.005	0.999
14	431.67	lnWT+IUCN	12.717	577.75	0.001	1.000
15	432.90	WT+lnSCI+IUCN	13.952	1070.33	0.000	1.000
15	433.63	lnWT+lnSCI+IUCN	14.678	1538.64	0.000	1.000
14	440.94	lnSCI+lnIUCN	21.999	59713.85	0.000	1.000

Other predictors: WT = species body mass, SCI = Safari Club International trophy score. IUCN = International Union for the Conservation of Nature classification for species. ‘ln’ = natural log. Evidence ratio is the likelihood of a model being the ‘best’ model compared with the top ranked model. All main effects models with transformed and untransformed predictors used.

**Table 2:** Predictors of bovid trophy value.

Predictor	R^2^ entered second	R^2^ entered third	Parameter estimate (CI) full model	Parameter estimate (CI) reduced model, fig. 2
Rarity (IUCN)	14.3%	9.8%	−0.89 (−1.13∶−0.63)	−1.07 (−1.41∶−0.84)
Body Mass	28.7%	1.2%	0.17 (0.03∶0.33)	0.39 (0.31∶0.47)
Trophy Index (SCI)	30.1%	2.1%	0.0028 (0.0010∶0.0046)	0.0050 (0.0040∶0.0060)

Partial r-squared values (%). All models with country ID entered first. Parameter estimates from full model (ranked first in table 1, and from models with country plus single predictor.

The relationships using each taxon value averaged among countries, and therefore a single datum per taxon, were similar. The exponent for body mass was less steep than that within country (0.39, CI 0.24 to 0.51), while that for IUCN category and IUCN index were similar (slope = −1.03 CI −1.53 to −0.47) and 0.005, CI 0.0034 to 0.0066 respectively). The best models were those including SCI index and IUCN score (*w* = 0.25), and the model also including (log) body mass (*w* = 0.18).

### Felidae

Seven species of cat are hunted and recorded as trophies by the SCI in Africa. One of these (the African golden cat, *Profelis aurata*) is hunted rarely, and does not appear in our data set. Where hunted, lioness are priced separately, and we treat them as distinct entities here. (The SCI do not list lioness, hence they do not have an SCI score).

The power law model predicting trophy value as a function of body mass was clearly best (*w* = 0.82), with slope consistent with proportionality (1.001, CI: 0.85 to 1.15. Body mass is highly correlated with SCI index (r = 0.91), and SCI index does not add usefully to a prediction based on body mass. Price mass relationships within countries were also consistent with proportionality; slopes on the log-log scale vary between 0.95 and 1.13.

## Discussion

The value of hunted individuals was related to both the characteristic trophy size of the species (which is highly correlated with body size) and to the perceived rarity and availability of the species, as advertised by IUCN rating. Less easily quantified attributes of species were also influential; the qualitative hunting notes of the SCI are suggestive of a ‘charisma’ effect, particularly for comparisons between families. The size effect is also likely to be attributable to charisma to some extent. Felidae and Rhinoceridae, for example, were on average more expensive to hunt than predicted by either size or IUCN class. While our observations are representative only of the consumers these leaflets were aimed at, this variation is related to widely held attitudes, based on anthropomorphic responses to the perceived ‘lifestyle’ of the animal [13]. There was no clear predator effect. Some predators (felids) were highly valued, while others (canids, hyaenas) were not, and this accords to some extent with reputation, linked to perceived pest status, as reported by Kellert [11]. This underlines the importance of aesthetic responses [16]. Operators use the ‘big five’ concept to market lion, leopard, buffalo, rhino and elephant to trophy collectors.

Perceived rarity did have a clear effect; the price of hunting bovid species increased with IUCN class. The categories are ordinal, so the inter-category intervals were not easily interpretable, but if category affects the availability of licenses, the potential for an AAE clearly exists, as for the hunted caprinids described by Courchamp et al. [4]. For this potential to be realized, two conditions need to be fulfilled. First, a category change (higher up the rarity/risk axis) is required to increase demand. Second, this increase needs to be large enough to provide an incentive for more licenses to be issued. If responsible management reacts to a higher IUCN category by reducing license numbers, the effect will not be realized. Under the former circumstances, trophy hunting could become unsustainable. A further assumption is that a change in status within a taxon results in an effect similar to that between taxa with the same status difference. Also, for many species, only a minority of adults carry trophies, so the increased demand for this subset of the population will have more subtle effects on the population.

Price increments between IUCN categories provide some basis for assessing the effect on demand. Those between ‘least concern’ and ‘least risk’ and between ‘near threatened’ and ‘vulnerable’ are considerable (figure 2c), and may be large enough to provide the necessary incentive. As Courchamp et al. [4] point out, declaring rarity in the absence of protection is potentially dangerous, and this may apply particularly to species where demand is elastic (and where quota-setting is not guided by science).

Bovid prices increased less than proportionately with size, in contrast with felids, where the exponent was close to 1.0, and was consistent among countries. Trophy size was a better signal of quality (price) than body size. Further evidence that charisma is linked to value is provided by hunter preferences as reported by Lindsey's (2006) study. Hunters were asked which species they were ‘keen’ to hunt. We examined the prices of the bovid species named by Lindsey's hunters, and compared them with a prediction based on country, trophy size and rarity. We found that most of those listed tended to be more expensive than predicted. This was particularly clear for buffalo, sable and roan. These tend to be species attracting similar comments to felids in the SCI hunting remarks. The buffalo is described as an ‘excellent game animal’ and a ‘major trophy’ and the eland is described as ‘one of the great trophies of Africa’ and an ‘outstanding game animal’ [14].

The benefit of hunting to conservation could be maximized by taking account of how size and rarity combine to influence value, particularly if co-operation among countries for license availability can be achieved. A crucial aspect of trophy hunting is that the effect of rarity on sustainability is modulated through the response of the rights holders. Given the evidence for an effect of IUCN status on demand, a downward adjustment in license numbers in response to a change in IUCN status need not lead to a loss of revenue to conservation in any country, provided other countries offering the species raise license prices similarly. For lions, where trophy hunting can account for a high proportion of mortality [17], increases in demand caused by perceived rarity could be particularly influential. Conservationists considering change in IUCN category should take into account both the possible increase in demand, and the likely reaction in different countries to that increase. Price increases for lions have been observed in response to the threat of CITES sanction and reduced quotas in some countries; in western Zimbabwe, where a hunting ban was imposed in 2005, the price of a lion trophy has risen from USD 4,000 in 2005 with one operator just before the reduction, to USD 30,000 with the same operator in 2009. The effect of size and less tangible charisma effects on price suggest that an optimal adjustment to licensing may differ significantly between taxonomic groups.

## Materials and Methods

We used 147 pamphlets displayed by hunting operators exhibiting at the 2004 Safari Club International (SCI) convention (Reno, USA). All pamphlets seen advertising African hunting safaris (countries in table 1) were collected. The prices advertised to hunt each species were extracted. Some operators trade in different areas within a country and in different countries; we derived the average price among operators in each country as our best estimate of the price for the taxon in that country.

The data were derived from 10 countries across Sub-Saharan Africa. A total of 159 taxa from 18 families were offered as quarry. Within the Bovids, operators tended to define taxa not formally species, usually geographic sub-species. We therefore used common names to differentiate taxa. Bovids dominated our data set (72.3% of prices used).

Predictors (country, rarity and size) were, as expected, inter-correlated: for example, the species available differs among countries. We therefore used sequential GLM models to assess the effect size of each predictor in influencing price. In sequential models, the effect of each predictor is adjusted only for the terms preceding it [18]. Models were compared differing in both predictors and in the scale of their measurement. Species body mass followed Kingdon [19]. To index rarity/availability we used IUCN categories, after [4]. These were used as an ordinal index. (In order of descending risk: V = vulnerable, LR/NT = Least risk, near threatened, LR/CD = Least risk, conservation dependent, LR/LC = Least risk, least concern). The country where the trophy was offered was entered to all models, given established price differences [2]; country identity was entered first to each model as a fixed blocking factor.

For bovids, we also used the SCI score. This is used by the hunting industry to compare trophy sizes *within* a species [14]. Species trophies are measured differently depending of the shape of the horns (if present) and other body parts. In Suidae, Elephantidae and Hippopotimidae, which lack horns, the SCI use metrics of tusk size. SCI indices can therefore be compared only within families. The SCI index for bovids depends on the shape and size of horns; it may be an aggregate of linear measures and circumferences (the dominant methodology) or it may use the span between horns (as for buffalo). The index is closely correlated with body size (R-squared in the model predicting body mass from SCI index with both on a log scale = 84.0%, slope = 1.47, CI = 1.35–1.59). Trophy indices increase disproportionately with body size (the CI lies clearly above 1.0). But some taxa plainly have trophies that are distinctly larger than predicted by body mass, and *vice versa*. Several of the gazelles and kudu fall into the former category, while the buffalo, reedbuck and duiker tend to have smaller trophies compared with a prediction based on their size. The use of this index allowed us to explore how the value of a trophy animal is a function of trophy size compared with body size.

Testing different scales of x allowed us to assess *how* value changes with our predictors. If an exponential model (y = a.e^bx^, fitted as log(y) = b.x) fits, an increment in *x* of the same magnitude results in a fixed percentage increase in *y*. A model of the form y = a.x^b^ (fitted by regressing log(*y*) on log(*x*)) fits a power law, allowing the gradient of y versus x to vary along the x axis. The form of these functions supports different explanations of price variation.

We used multimodel inference to compare models [20], [21]. Models were constructed using the SAS MIXED procedure [22]. The SAS COMPMIX macro (http://www.stat.wisc.edu/~yandell/software/sas/compmix.sas) was used to compute model weights and AIC_C_ values. Model weights (*w*) give the probability for each model that it is the best fit among those compared. All models used log-price (USD) as response variable. Residuals were approximately normally distributed and invariant across the range of fitted values. The GLM procedure was used to compute R-squared values (giving the amount of variation in price explained) for models and predictors.

We looked at patterns both among and within families. While taxa are unevenly represented across families, a family level exploration reveals the effect of gross morphological differences on pricing. Size and rarity might be expected to be more important within families.
